# Impact of a custom-made 3D printed ergonomic grip for direct laryngoscopy on novice intubation performance in a simulated easy and difficult airway scenario—A manikin study

**DOI:** 10.1371/journal.pone.0207445

**Published:** 2018-11-20

**Authors:** Sung-Hoon Kim, Jeonghoon Kwon, Youn-Jung Kim, Hyung-Joo Lee, Hyo-Chang Seo, Seung Bok Lim, Segyeong Joo, Dong-Woo Seo, Won-Young Kim, Sang-Bum Hong

**Affiliations:** 1 Department of Anesthesiology and Pain Medicine, Asan Medical Center, University of Ulsan College of Medicine, Seoul, South Korea; 2 Department of Biomedical Engineering, Asan Medical Center, University of Ulsan College of Medicine, Seoul, South Korea; 3 Department of Emergent Medicine, Asan Medical Center, University of Ulsan College of Medicine, Seoul, South Korea; 4 Emergency Nursing Department, Asan Medical Center, Seoul, Korea; 5 Department of Biomedical Informatics, University of California San Diego, School of Medicine, San Diego, United States of America; 6 Department of Pulmonology, Asan Medical Center, University of Ulsan College of Medicine, Seoul, South Korea; Imam Abdulrahman Bin Faisal University College of Medicine, SAUDI ARABIA

## Abstract

Direct laryngoscopy using a Macintosh laryngoscope is the most widely used approach; however, this skill is not easy for novices and trainees. We evaluated the performance of novices using a laryngoscope with a three-dimensional (3D)-printed ergonomic grip on an airway manikin. Forty second-year medical students were enrolled. Endotracheal intubation was attempted using a conventional Macintosh laryngoscope with or without a 3D-printed ergonomic support grip. Primary outcomes were intubation time and overall success rate. Secondary outcomes were number of unsuccessful attempts, first-attempt success rate, airway Cormack-Lehane (CL) grade, and difficulty score. In the easy airway scenario, intubation time, and the overall success rate were similar between two group. CL grade and ease-of-use scores were significantly better for those using the ergonomic support grip (*P* < 0.05). In the difficult airway scenario, intubation time (49.7±37.5 vs. 35.5±29.2, *P* = 0.013), the first-attempt success rate (67.5% vs. 90%, *P* = 0.029), number of attempts (1.4±0.6 vs. 1.1±0.4, *P* = 0.006), CL grade (2 [2, 2] vs. 2 [1, 1], *P* = 0.012), and ease-of-use scores (3.5 [2, 4] vs. 4 [3, 5], *P* = 0.008) were significantly better for those using the ergonomic support grip. Linear mixed model analysis showed that the ergonomic support grip had a favorable effect on CL grade (*P*<0.001), ease-of-use scores (*P*<0.001), intubation time (*P* = 0.015), and number of intubation attempts (*P* = 0.029). Our custom 3D-printed ergonomic laryngoscope support grip improved several indicators related to the successful endotracheal intubation in the easy and difficult scenario simulated on an airway manikin. This grip may be useful for intubation training and practice.

## Introduction

Endotracheal intubation via direct laryngoscopy is a critical, life-saving procedure for securing patient airways in various clinical settings. However, this skill could be difficult for novices and trainees to master [1]. In particular, airway management for patients with difficult airways remains a challenging issue in teaching hospitals, and considerable learning time is required before this technique can be used in clinical practice. Although various types of video laryngoscopy are now widely accepted airway management techniques [2–5], conventional Macintosh laryngoscopy is still the most selected choice.

Assessing medical instruments based on an ergonomic concept is essential. Differences in shape and details can significantly affect muscle load and performance[6]. Three-dimensional (3D) printed models enable physicians and engineers to consider ergonomic concerns and design alternatives in a timely and cost-effective manner. However, information about the application of 3D-printing in the field of anesthesiology or emergency medicine is sparse. We hypothesized that ergonomic support would make endotracheal intubation easier and quicker for novice practitioners by providing a better glottic view. Herein we evaluated the performance of a laryngoscope with an ergonomic grip on an airway manikin by novice practitioners.

## Methods

The current prospective, randomized, controlled, open-label study was performed in the simulation center of a tertiary care facility and was approved by The Institutional Review Board of Asan Medical Center (approval number 2016–0366). Written informed consent was waived.

### Study design and participants

Participants were recruited from among the second-year medical students; totally, 40 students were enrolled. None of the enrolled students had a previous experience with endotracheal intubation. Each student was given a standardized 20-min oral presentation, followed by a demonstration of the endotracheal intubation technique. A written description of how to use the laryngoscope and how to determine the Cormack-Lehane (CL) grade of the airway was also given to each participant [7]. The current study was performed at Asan Medical Center Simulation Center between July and August 2016. After the instructional session, participants performed sequential intubations on the airway manikin under two different scenarios: a normal airway in the supine position (easy scenario) and a difficult airway (difficult scenario) performed on a Laerdal Airway Management Trainer (Laerdal, Norway). To limit the mobility of the neck and opening of the mouth, a rigid Philadelphia cervical collar (Philadelphia Cervical Collar Co., Thorofare, NJ, USA) was applied. As there were two airway devices and two scenarios, four different device-scenario combinations were possible and randomized among the 40 participants.

The primary outcomes included intubation time and overall success rate. Intubation time was defined as the time between the tip of the laryngoscope blade passing the manikin’s teeth to the first observed chest expansion using the resuscitation bag. The investigator verified the position of the endotracheal tube after each intubation attempt by directly viewing the trachea. Secondary outcomes included the number of intubation attempts, first-intubation attempt success rate, airway CL grade, and difficulty score. Those laryngeal views were graded according to the modified CL classification by referring to an illustration [8]. Intubation was halted if the tip of the blade came out of the manikin’s mouth. A failed attempt was defined as either an intubation that took longer than 120 seconds or the insertion of the tube into the esophagus[9]. Endotracheal intubation was deemed a failure if the participant did not succeed after three attempts in a total of 120 seconds[10]. The overall ease of tracheal intubation was reported using a verbal rating scale scored from 1 (very hard) to 5 (very easy).

### Fabrication of the 3D-printed laryngoscope grip

**Fig 1:**
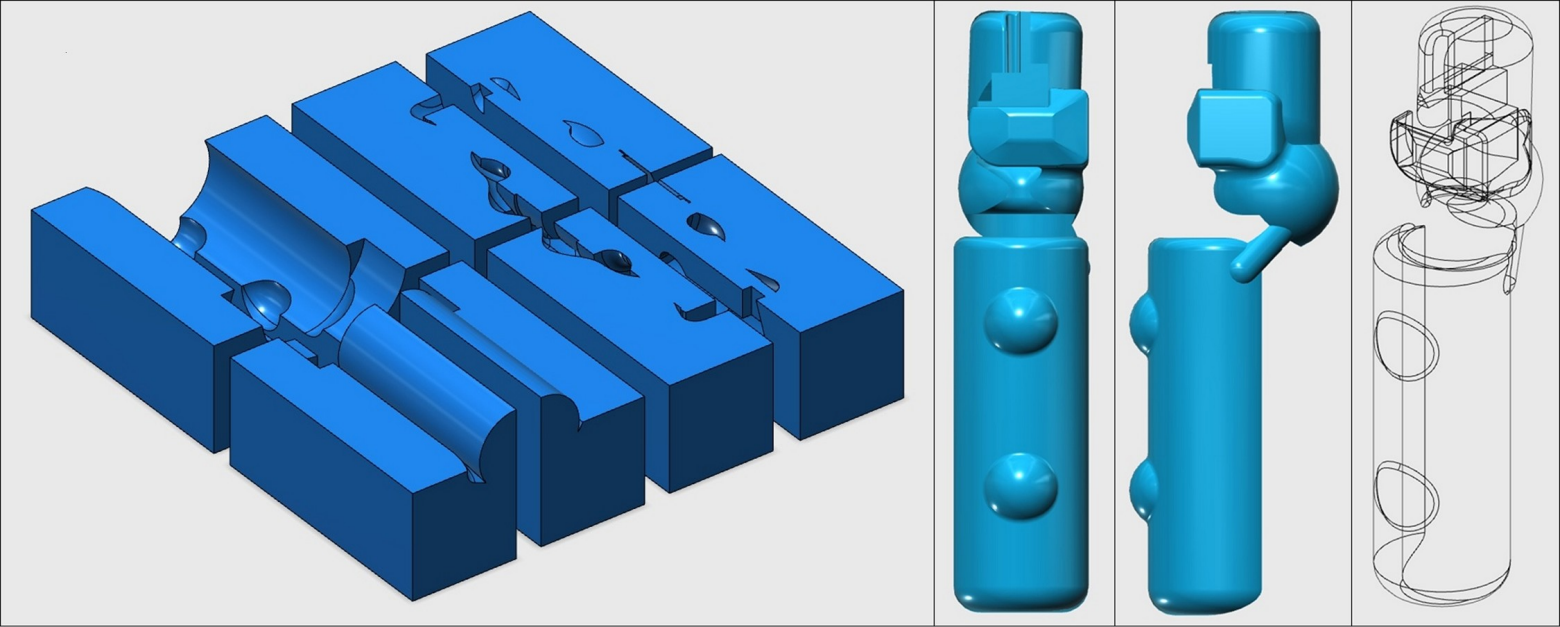
Designed ergonomic laryngoscope grip model.

**Fig 2:**
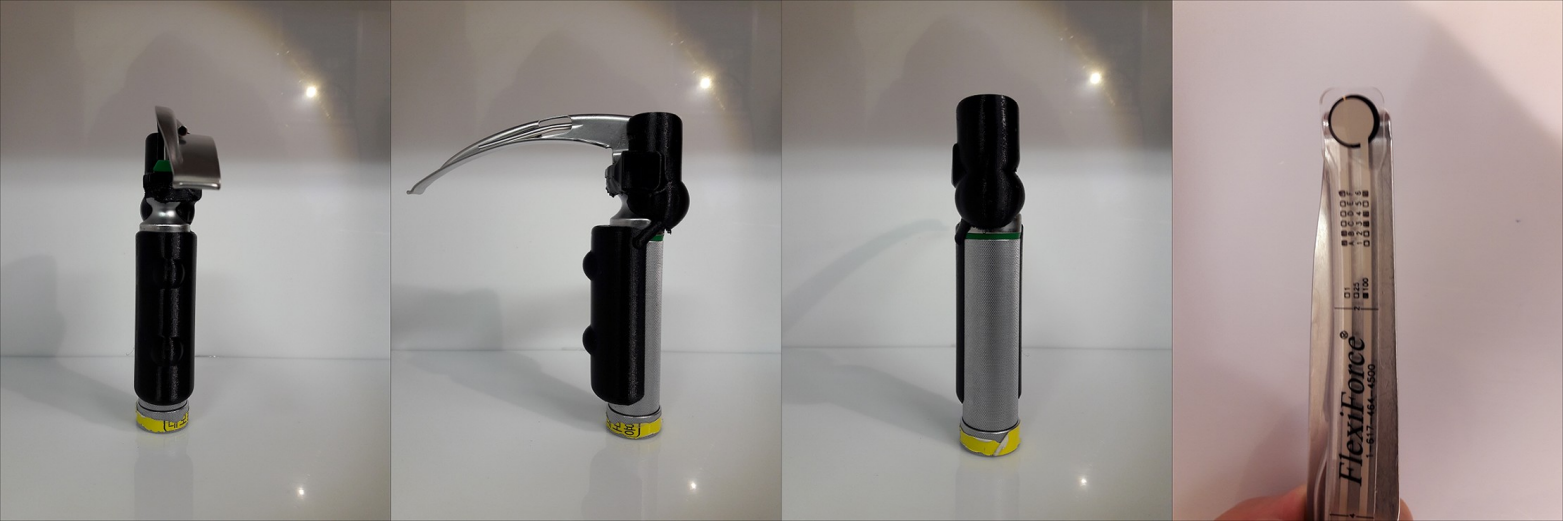
3D-Printed ergonomic grip applied to the Macintosh laryngoscope.

**Fig 3:**
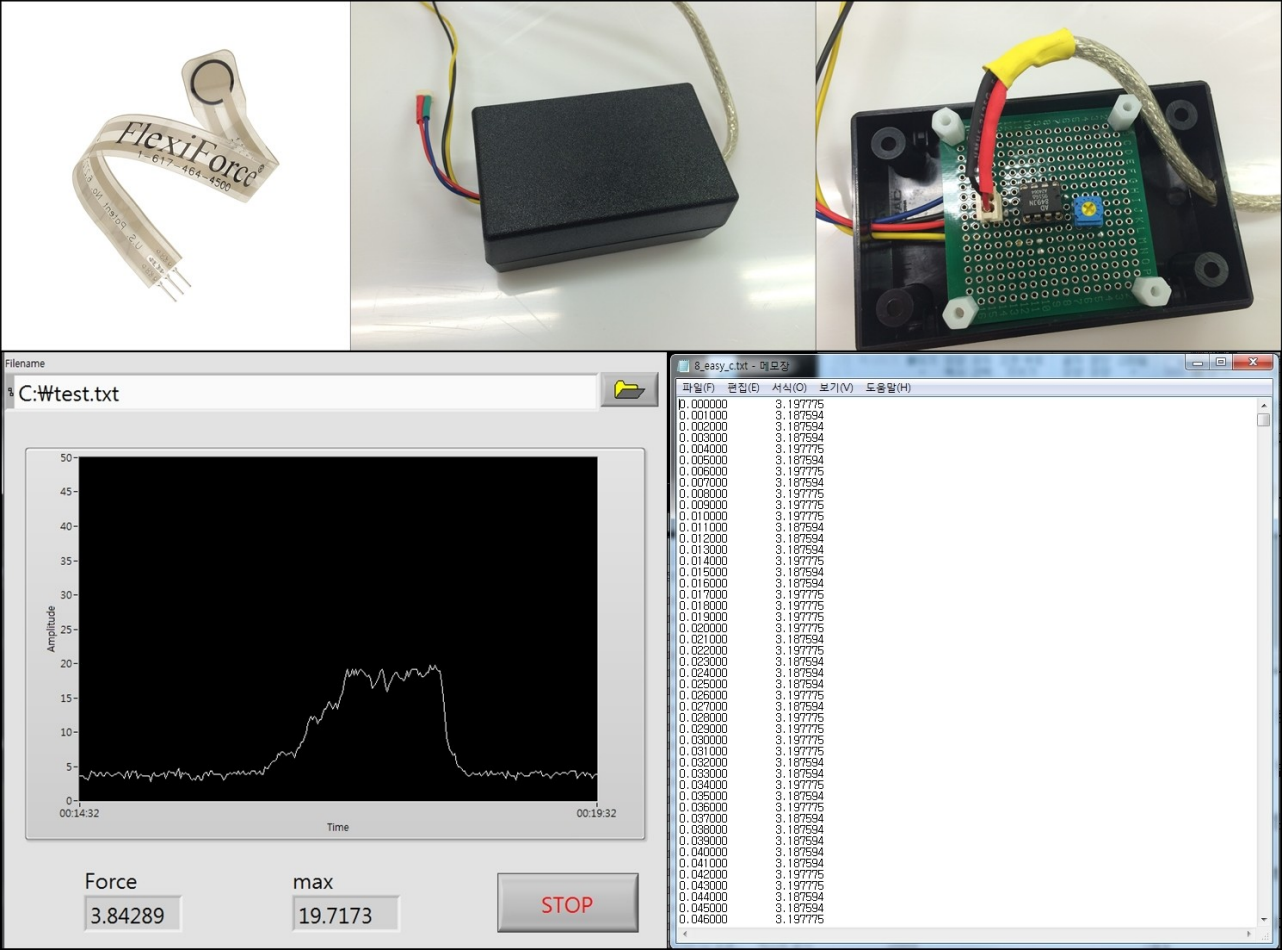
Peak force measurement during intubation using a FlexiForce standard load/force sensor and LabView programming.

Firstly, a digital 3D-model of the grip was designed (Fig 1) using the 123D Design software (Autodesk, USA). Then, the final design file was converted to be compatible with a Cubicon Single 3D-printer (HighVision, Korea), which used a soft and elastic thermoplastic polyurethane filament to print the designed grip. The 3D-printed grip was then applied to a Macintosh laryngoscope (Fig 2). To measure the peak force during intubation, a FlexiForce standard load/force sensor (Tekscan, Boston, MA, USA) was applied to the tip of the blade (Fig 3). All intubations were performed with a 7.5-cuffed endotracheal tube (Mallinckrodt TaperGuard Oral/Nasal Tracheal Tube, Covidien, MA, USA) and a malleable plastic stylet bent with a J-shaped curvature. Endotracheal intubation was attempted by each novice using a conventional Macintosh laryngoscope with or without the 3D-printed ergonomic support grip. A blade size of 3 was used for each device.

### Statistical analysis

A previous study reported that the standard deviation (SD) in intubation time taken by medical students was 18 seconds[11]. At least 38 subjects were required to detect a difference in mean intubation times of 12 seconds with an alpha error of 0.05 and a power of 0.8. We enrolled 40 participants in our study to minimized data loss. All data are presented as the mean ± SD, median (interquartile range), or numbers. Normal distribution was assessed using the Kolmogorov-Smirnov test. Pairwise version of t-test or the Mann-Whitney U test was used for continuous variables. The chi-square test or the Fisher’s exact test was used for the comparison of success rate with the first intubation attempt between the groups. To evaluate factors affecting CL grade, ease-of-use score, intubation time, and number of attempts, a linear mixed model with a first-order autoregressive covariance matrix was used. All statistical data were analyzed using SPSS 21.0 (IBM Corp., Armonk, NY, USA) and R version 3.1.2 (R Foundation for Statistical Computing, Vienna, Austria). *P*-values of < 0.05 were considered significant.

## Results

**Fig 4:**
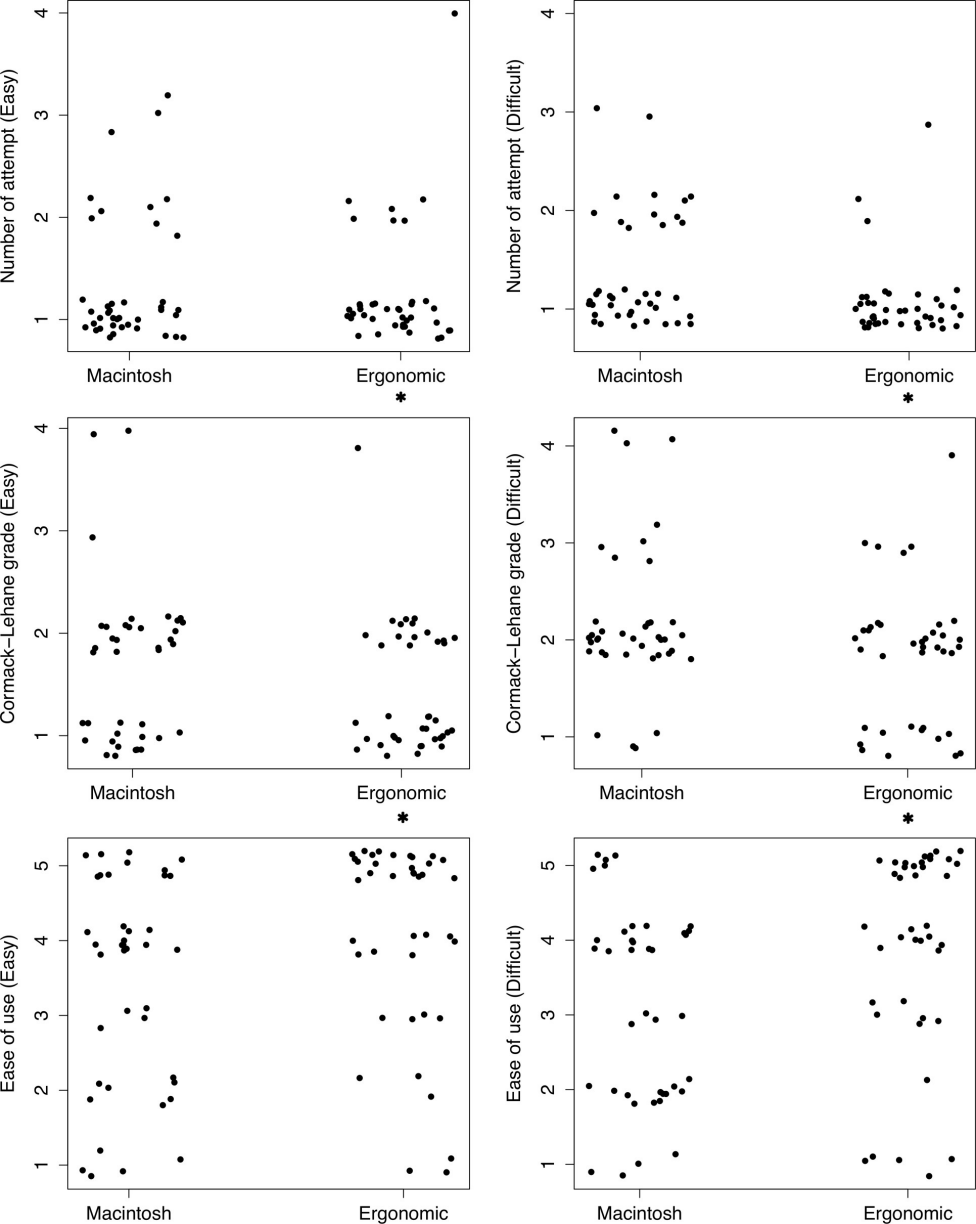
Number of intubation attempts, Cormack-Lehane grade, and ease-of-use scores for intubation groups with and without the ergonomic support grip for easy (left) and difficult (right) airway scenarios; *P < 0.05.

A total of 40 novice practitioners (second-year medical students; 13 females and 27 males) were enrolled. All participants performed four intubation attempts in each scenario (easy and difficult), and no data were excluded. In the easy scenario, intubation time, the overall success rate, first-attempt success rate, number of attempts, and peak force during intubation were similar between those using the conventional Macintosh laryngoscope with and without the ergonomic support grip (Table 1). The CL grade and ease-of-use score were significantly better for the ergonomic support group than for the conventional laryngoscope group (*P* < 0.05). In the difficult scenario, intubation time was significantly shorter for those in the ergonomic support group than for those in the conventional group (35.5 ± 29.2 versus 49.7 ± 37.5, *P* = 0.013). Other performance variables, including intubation success with the first attempt (90.0% versus 67.5%, *P* = 0.029), intubation attempts (1.1 ± 0.4 versus 1.4 ± 0.6, *P* = 0.006, Fig 4), CL grade (1.9 ± 0.7 versus 2.2 ± 0.7, *P* = 0.010, Fig 4), and ease-of-use score (3.9 ± 1.4 versus 3.1 ± 1.3, *P* = 0.005, Fig 4), were also improved for those using the ergonomic support grip than for those not using it. Peak force during intubation in the difficult scenario was not different between both groups.

**Table 1:** Performance data for novices using a Macintosh laryngoscope with and without the ergonomic support grip for easy and difficult airway scenarios.

	Macintosh laryngoscope	Ergonomic support grip	P-value
Easy scenario			
Intubation time (s)	48.2 ± 35.7	41.3 ± 31.8	0.239
Intubation success rate (%)	87.5%	92.5%	0.712
Intubation success at first trial (%)	75% (30/40)	82.5% (33/40)	0.582
Intubation attempt (n)	1.3 ± 0.6	1.2 ± 0.6	0.421
Peak force during intubation (N)	2.2 [1.65, 4.94]	2.75 [1.45, 4.39]	0.662
Cormack-Lehane grade (1–4)	2 [1, 2]	1[1, 2]	0.034
Ease-of-use score (VRS; 1–5)	4 [2, 5]	5[3.25, 5]	0.009
Difficult scenario			
Intubation time (s)	49.7 ± 37.5	35.5 ± 29.2	0.013
Intubation success rate (%)	87.5%	92.5%	0.712
Intubation success at first trial (%)	67.5% (27/40)	90% (36/40)	0.029
Intubation attempt (n)	1.4 ± 0.6	1.1 ± 0.4	0.006
Intubation time (s)	49.7 ± 37.5	35.5 ± 29.2	0.013
Peak force during intubation (N)	2.75 [1.7, 3.8]	3.16 [2.2, 4.7]	0.619
Cormack-Lehane grade (1–4)	2 [2, 2]	2 [1, 2]	0.012
Ease-of-use score (VRS; 1–5)	3.5 [2, 4]	4 [3, 5]	0.008

Data are presented as the median [interquartile range], mean ± standard deviation, or numbers. Up to 3 intubation attempts were counted within 2 min. VRS, verbal rating scale.

Using a linear mixed model with a first-order autoregressive covariance matrix, the following formulas were derived to predict the CL grade, ease-of-use score, intubation time, and number of attempts (Table 2):

**Table 2:** Linear mixed model for predicting Cormack-Lehane grade, ease-of-use score, intubation time, and number of intubation attempts.

Outcome	Variables	Estimate	Lower	Upper	P-value
Cormack-Lehane	Sex	0.210	−0.114	0.535	0.199
	Easy	−0.428	−0.646	−0.209	<0.001
	3D	−0.309	−0.479	−0.139	0.001
	Intercept	2.102			
Ease-of-use score	Sex	−0.001	−0.569	0.566	0.996
	Easy	0.307	−0.111	0.725	0.148
	3D	0.662	0.322	1.001	<0.001
	Intercept	3.171			
Intubation time	Sex	−4.388	−20.370	11.595	0.583
	Easy	0.464	−9.599	10.528	0.927
	3D	−9.431	−17.008	−1.853	0.015
	Intercept	49.502			
Intubation attempt	Sex	0.024	−0.196	0.244	0.828
	Easy	0.033	−0.141	0.208	0.706
	3D	−0.166	−0.314	−0.018	0.029
	Intercept	1.312			

CL grade = 2.102 − 0.210 (female) − 0.428 (easy) − 0.309 (ergonomic)

Ease-of-use score = 3.171 − 0.001 (female) + 0.307 (easy) +0.662 (ergonomic)

Intubation time = 49.502 − 4.388 (female) + 0.464 (easy) − 9.431 (ergonomic)

Intubation attempts = 1.312 + 1.312 + 0.024 (female) + 0.033 (easy) − 0.166 (ergonomic)

The model showed that the ergonomic support grip had a favorable effect on CL grade (*P*<0.001), ease-of-use scores (*P*<0.001), intubation time (*P* = 0.015), and number of intubation attempts (*P* = 0.029).

## Discussion

Our study demonstrated that a 3D-printed ergonomic support grip for a conventional Macintosh laryngoscope facilitates greater success with endotracheal intubation by novice practitioners on an airway manikin, particularly as the level of airway difficulty increased. In the difficult scenario, first intubation attempt success rates were significantly higher for those using the 3D-printed ergonomic support grip, and other performance variables, including the intubation time, number of intubation attempts, CL grade, and ease-of-use score, were superior in ergonomic support grip group. Furthermore, the linear mixed model statistically confirmed that the application of our ergonomic support grip had a favorable effect on the CL grade, ease-of-use score, intubation time, and number of intubation attempts. We also provided the entire file on the 3D-printed ergonomic support grip for the reproducibility of this study. To the best of our knowledge, this is the first study to apply the 3D-printing technology to direct laryngoscopy.

While we found no significant difference between the groups in overall and first-attempt success rates or intubation time with the easy scenario, most performance variables were improved with the use of the ergonomic laryngoscope support grip in the difficult scenario. First attempt success rates are an important factor to consider because the number of intubation attempts ultimately affects the overall success rate and repeated attempts increase the risk of further oropharyngeal injury, hemodynamic instabilities, and hypoxemia[12]. A review by Mihai *et al*. reported that the first intubation attempt success rate for difficult airways was approximately 92.3% when using Glidescope, which has been studied relatively recently[13]. Similarly, our study shows that the ergonomic support grip affords a higher first-attempt and overall success (90.0% and 92.5%, respectively) rate with difficult airways. Although performance variables for both groups were similar in the easy airway scenario, intubation times were shorter for those using the ergonomic support grip, with a mean time difference of 7 and 14 seconds between the groups in each scenario. As from Table 1, peak force registered with the 3D printed ergonomic grip was higher in both easy and difficult airway scenarios. This point could mean that the newly designed grip could modify the leverage fulcrum and allow for higher peak force. On the one hand, this might explain better performance, but once translated into clinical practice, this might result in a potential for trauma or increased peri-intubation patients’ stimulation, including the hemodynamic response [5,14,15].

The ergonomic grip we designed offers a high intubation success rate at first trial (90%) even in a difficult airway scenario. In the current study, all difficult airway scenarios were assessed after applying a rigid Philadelphia cervical collar to limit the mobility of the neck and opening of the mouth. However, 87.5% intubation success rate by a second year medical student who is inexperienced in airway management might not meet the threshold of a difficult airway. It is important to note that although the cervical collar is widely used to simulate difficult airway scenarios, it only produces a predicted scenario that may not accurately represent every difficult airway encountered. Thus, intubation success rates could be overestimated without validating the effect of the cervical collar. Further, the degree to which the cervical collar is applied may differ among practitioners and patients. This uncertainty and heterogeneity in simulating difficult airways make it difficult to evaluate and compare the performance of new airway devices including this study. However, we think that this study can be reproducible because airway manikins are widely used test equipment.

A trustworthy assessment of the ergonomic quality of a device is possible through working tests that include both subjective ratings and necessary objective physiological measurements. The lowering cost of 3D-printing has made the use of this technology widespread, and the medical application of this technology is increasing as a result of its customization and personalization benefits. The medical applications of 3D-printing will expand personalized and precision medicine and build customized and personalized tools that will improve patient safety and outcomes.

Our study has several limitations. First, an airway manikin does not precisely reproduce intubation conditions in real patients; therefore, our findings cannot be directly applied to clinical situations. Second, more practice with a Macintosh laryngoscope with and/or without the ergonomic grip will improve success rates and shorten intubation times. However, we only assessed four intubation attempts per participant. It is estimated that approximately 50 attempts are needed to achieve a consistent success rate of >90% with a Macintosh laryngoscope[16,17]. More tests are needed to see the learning curves over time. Third, the carryover effect can occur because the participants perform the procedure sequentially. We tried to reduce the carryover effect by changing the order of use of the instrument. However, we think we could not entirely exclude the carryover effect. Fourth, we applied only one sensor on the tip of the blade for measuring force. Since one sensor could not cover the entire blade area, our result might affect by this. Lastly, our study was not blinded as we could not hide which laryngoscope was being used from the participant or the investigator measuring the intubation time. Nonetheless, we believe that this did not significantly affect our results considering our clearly defined, robust endpoints.

In conclusion, our custom 3D-printed ergonomic laryngoscope support grip improved several indicators related to the successful endotracheal intubation in the easy and difficult scenario simulated on an airway manikin. Our 3D-printed ergonomic support grip will be useful for facilitating difficult airway management, and further clinical applications and studies are necessary to confirm our results in live patients.
